# Double-layer sign of the anterior lens capsule during cataract surgery: A case report

**DOI:** 10.1097/MD.0000000000041011

**Published:** 2025-01-17

**Authors:** Ruoxi Liu, Hongzhan Shao, Xiaoli Xu, Shanshan Huang

**Affiliations:** a Heilongjiang University of Chinese Medicine, The Second Affilated Hospital of Heilongjiang University of Chinese Medicine, Harbin, Heilongjiang, China; b Apex Eye Care Hospital, Nanyang, Henan, China; c Locomotive Hospital, Dalian, Liaoning, China; d Zhuhai People’s Hospital, Zhuhai, China.

**Keywords:** anterior lens capsule, case report, cataract, continuous curvilinear capsulorrhexis, double-layer

## Abstract

**Rationale:**

The double-layer sign of the anterior lens capsule during continuous curvilinear capsulorrhexis (CCC) in cataract surgery is a rare phenomenon. This case report highlights the occurrence of this sign and provides a practical technique for managing it.

**Patient concerns:**

A 55-year-old Chinese woman presented with blurred vision in her left eye. She had a history of nuclear sclerotic cataracts, but no abnormalities of the anterior capsule were noted in her preoperative evaluation.

**Diagnoses:**

Nuclear sclerotic cataract in the left eye with no preoperative abnormalities of the anterior lens capsule.

**Interventions:**

During CCC, a double-layer sign of the anterior lens capsule was identified. The surgeon successfully managed the double-layer sign by simultaneously grasping both layers with capsulorhexis forceps to complete CCC without complications.

**Outcomes:**

Postoperatively, the patient achieved 20/20 vision in the treated eye, and no complications were observed.

**Lessons:**

The double-layer sign of the anterior lens capsule is a rare finding during cataract surgery. Although its exact cause remains unclear, the technique described offers a practical approach to manage this intraoperative challenge effectively.

## 1. Introduction

The occurrence of a double-layer sign of the anterior lens capsule during continuous curvilinear capsulorrhexis (CCC) in cataract surgery has rarely been reported.^[1–7]^ Recently, we encountered a patient exhibiting this sign during cataract surgery. Recently, we encountered a patient exhibiting this sign during cataract surgery. Here, we report the surgical process and management techniques used, hoping they will serve as a useful reference for our colleagues in ophthalmology.

## 2. Case report

A 55-year-old Chinese woman presented with blurred vision in her left eye in June 2024. She had undergone cataract surgery in her right eye 3 years prior. Her past medical history was otherwise unremarkable: she had no history of heat exposure, ocular trauma, or uveitis. Her family history for ocular disease was also unremarkable. On initial examination, her best-corrected visual acuity was 20/100 in the left eye and 20/20 in the right eye. Intraocular pressure was 13 mm Hg in the left eye and 11 mm Hg in the right eye. Both corneas appeared clear. Nuclear sclerotic cataracts were present in the left eye. The anterior capsule appeared normal in the left eye, with no pseudoexfoliation noted. The fundus examination of both eyes was ophthalmoscopically normal. The classification of this patient’s cataract before surgery is 50% cortical opacity, II nuclear opacity.

**Figure 1.**
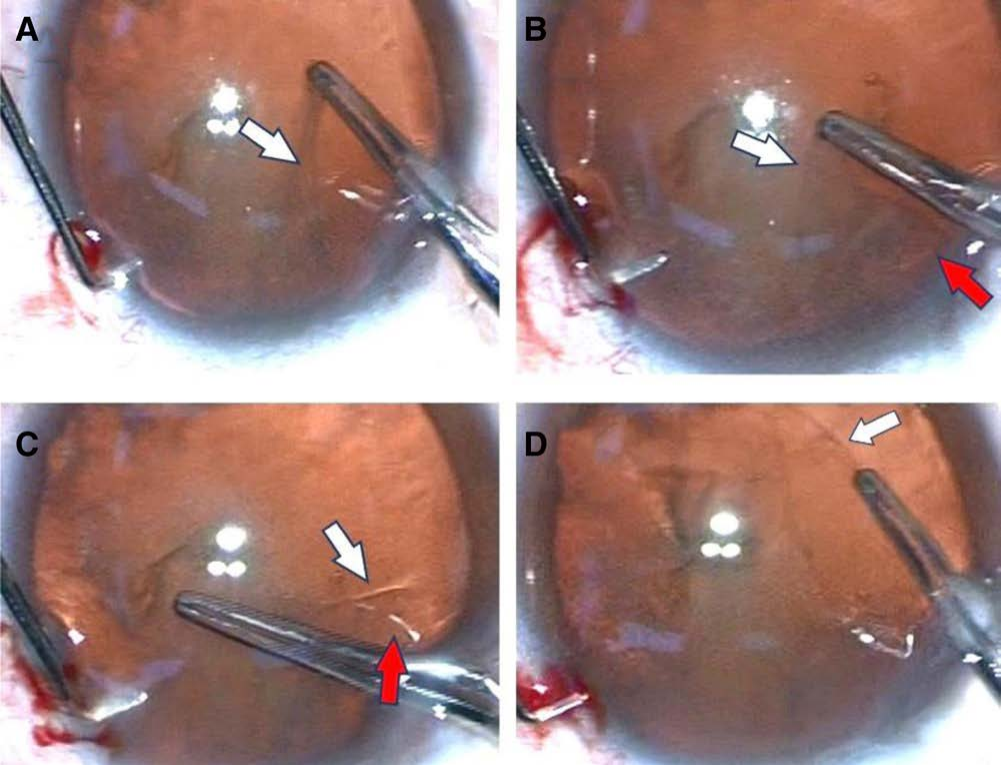
The double-layer sign of the lens capsule was visible during continuous curvilinear capsulorrhexis. In images A–C, the areas indicated by the white and red arrows show the double-layer sign of the anterior capsule. In image D, the capsulorhexis forceps are shown simultaneously grasping both layers of the anterior capsule to continue the continuous curvilinear capsulorrhexis.

The patient underwent phacoemulsification cataract extraction with implantation of a foldable acrylic lens in her left eye on June 14, 2024. A CCC was performed with capsulorhexis forceps. During capsulorrhexis, the anterior lens capsule appears a double-layer sign (Fig. 1). When the capsulorhexis reached the 12 o’clock position, a double-layer anterior capsule was observed (Fig. 1A–C). The surgeon then performed 2 additional capsulorhexis tears at the 4 o’clock position. Using capsulorhexis forceps, the surgeon grasped both layers of the anterior capsule simultaneously and continued with the continuous curvilinear capsulorhexis to complete the procedure (Fig. 1D). No radial tears occurred in the anterior capsule during the capsulorhexis. After completing the capsulorrhexis, phacoemusification and aspiration of residual cortex were performed uneventfully. The intraocular lens (Eyebright, A1-UV; Beijing, China) was implanted. The patient’s postoperative course was uneventful. Her right visual acuity was 20/20 on July 8, 2000.

## 3. Outcomes

The surgical procedure was successfully completed without any complications. The surgeon managed the double-layer anterior capsule by simultaneously grasping both layers with capsulorhexis forceps during CCC. This approach effectively prevented radial tears and allowed the capsulorrhexis to proceed smoothly.

Postoperative observations revealed no signs of capsular instability or zonular damage. The patient’s visual acuity in the left eye improved significantly, reaching 20/20 by the follow-up visit on July 8, 2024. The implanted intraocular lens remained well-centered, and there were no signs of postoperative complications such as posterior capsule opacification, inflammation, or increased intraocular pressure.

The patient expressed satisfaction with the surgical outcome, particularly noting the improved visual clarity and absence of discomfort. This positive outcome underscores the effectiveness of the described technique in addressing the challenges posed by the double-layer anterior capsule.

## 4. Discussion

The occurrence of a double-layer anterior capsule is a rare phenomenon in cataract surgery, and its cause remains unclear.^[1–7]^ In this case, no abnormalities of the anterior capsule were observed preoperatively. The double-layer sign of anterior capsule was only discovered during the continuous curvilinear capsulorhexis. Some studies have reported the possibility of tears occurring during capsulorhexis in cases with a double-layer anterior capsule.^[7]^ In this case, the surgeon achieved good results by using capsulorhexis forceps to simultaneously grasp both layers of the anterior capsule and proceed with the continuous curvilinear capsulorhexis. This technique may serve as a useful reference for ophthalmologists encountering similar cases.

However, this report is limited by its single-case nature, which restricts the generalizability of the findings. Additionally, the underlying causes of the double-layer anterior capsule were not investigated, as no abnormalities were observed preoperatively. Future studies should aim to identify potential risk factors or underlying mechanisms associated with this phenomenon to better inform preoperative assessments and surgical strategies. Despite these limitations, this report provides valuable insights and introduces a technique that may facilitate successful CCC in cases with a double-layer anterior capsule.

## Author contributions

**Conceptualization:** Ruoxi Liu, Xiaoli Xu, Shanshan Huang.

**Formal analysis:** Ruoxi Liu, Shanshan Huang.

**Investigation:** Ruoxi Liu, Xiaoli Xu.

**Methodology:** Ruoxi Liu.

**Project administration:** Ruoxi Liu, Hongzhan Shao.

**Resources:** Ruoxi Liu.

**Software:** Ruoxi Liu.

**Supervision:** Xiaoli Xu, Shanshan Huang.

**Validation:** Hongzhan Shao.

**Writing – original draft:** Ruoxi Liu.

**Writing – review & editing:** Ruoxi Liu, Shanshan Huang.
